# 2-((1*E*)-1-{2-[(2*Z*)-4-(4-Bromo­phen­yl)-3-phenyl-2,3-di­hydro-1,3-thia­zol-2-yl­idene]hydrazin-1-yl­idene}eth­yl)pyridin-1-ium bromide monohydrate

**DOI:** 10.1107/S160053681400347X

**Published:** 2014-02-22

**Authors:** Joel T. Mague, Shaaban K. Mohamed, Mehmet Akkurt, Ahmed T. Abd El-Alaziz, Mustafa R. Albayati

**Affiliations:** a Department of Chemistry, Tulane University, New Orleans, LA 70118, USA; b Chemistry and Environmental Division, Manchester Metropolitan University, Manchester M1 5GD, England; c Chemistry Department, Faculty of Science, Minia University, 61519 El-Minia, Egypt; d Department of Physics, Faculty of Sciences, Erciyes University, 38039 Kayseri, Turkey; e Kirkuk University, College of Science, Department of Chemistry, Kirkuk, Iraq

## Abstract

In the title hydrated molecular salt, C_22_H_18_BrN_4_S^+^·Br^−^·H_2_O, the aromatic rings make dihedral angles of 14.20 (12), 34.29 (10) and 68.75 (11)° with the thia­zole ring. In the crystal, mol­ecules are linked into chains running parallel to the *a* axis by association of the bromide ions and the water mol­ecules of crystallization with the cations *via* N—H⋯O, O—H⋯Br, C—H⋯N and C—H⋯Br hydrogen-bonding inter­actions. C—H⋯π and C—Br⋯π [3.7426 (11) Å, 161.73 (7)°] inter­actions are also observed, forming infinite chains extending along the *b*-axis direction.

## Related literature   

For general background to thia­zole compounds, see: Siddiqui *et al.* (2009 ▶); Quiroga *et al.* (2002 ▶); Hutchinson *et al.* (2002 ▶). For the biological activity of thia­zoles, see: Sharma *et al.* (2009 ▶); Ergenc *et al.* (1999 ▶); Bell *et al.* (1995 ▶); Patt *et al.* (1992 ▶); Jaen *et al.* (1990 ▶); Badorc *et al.* (1997 ▶); Rudolph *et al.* (2001 ▶). For structures with C—Br⋯π inter­actions, see: Jasinski *et al.* (2010 ▶); Zukerman-Schpector *et al.* (2011 ▶).
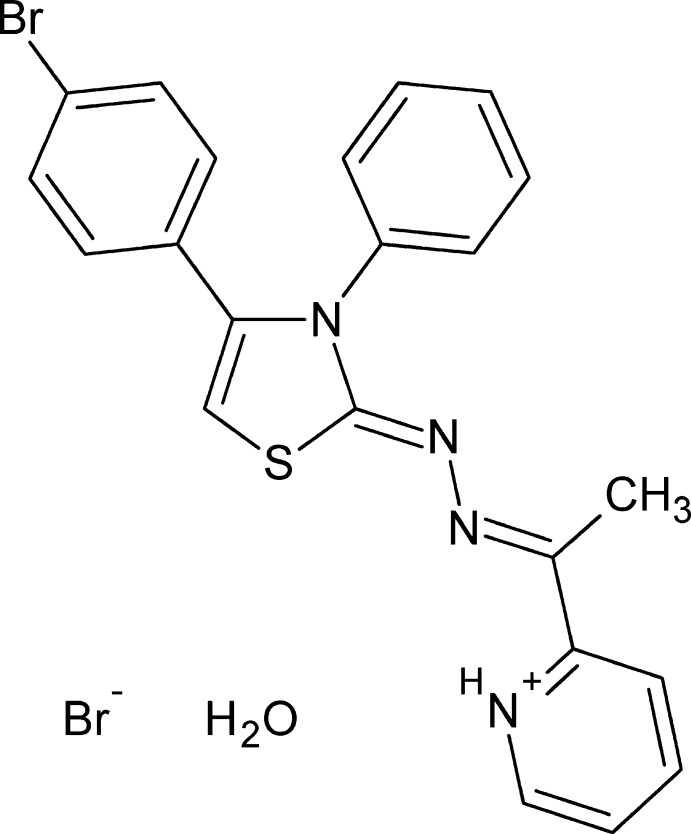


## Experimental   

### 

#### Crystal data  

C_22_H_18_BrN_4_S^+^·Br^−^·H_2_O
*M* *_r_* = 548.30
Triclinic, 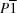

*a* = 5.5768 (6) Å
*b* = 9.2288 (9) Å
*c* = 22.574 (2) Å
α = 85.974 (1)°
β = 84.438 (1)°
γ = 79.000 (1)°
*V* = 1133.51 (19) Å^3^
*Z* = 2
Mo *K*α radiation
μ = 3.69 mm^−1^
*T* = 150 K
0.27 × 0.11 × 0.08 mm

#### Data collection  

Bruker SMART APEX CCD diffractometer
Absorption correction: numerical (*SADABS*; Bruker, 2013 ▶) *T*_min_ = 0.390, *T*_max_ = 0.760
20898 measured reflections
5870 independent reflections
4807 reflections with *I* > 2σ(*I*)
*R*_int_ = 0.038

#### Refinement  

*R*[*F* ^2^ > 2σ(*F* ^2^)] = 0.035
*wR*(*F* ^2^) = 0.095
*S* = 1.09
5870 reflections
272 parameters
H-atom parameters constrained
Δρ_max_ = 0.94 e Å^−3^
Δρ_min_ = −0.49 e Å^−3^

### 

Data collection: *APEX2* (Bruker, 2013 ▶); cell refinement: *SAINT* (Bruker, 2013 ▶); data reduction: *SAINT*; program(s) used to solve structure: *SHELXS97* (Sheldrick, 2008 ▶); program(s) used to refine structure: *SHELXL2013* (Sheldrick, 2008 ▶); molecular graphics: *ORTEP-3 for Windows* (Farrugia, 2012 ▶) and *DIAMOND* (Brandenburg & Putz, 2012 ▶); software used to prepare material for publication: *WinGX* (Farrugia, 2012 ▶) and *PLATON* (Spek, 2009 ▶).

## Supplementary Material

Crystal structure: contains datablock(s) global, I. DOI: 10.1107/S160053681400347X/qm2104sup1.cif

Structure factors: contains datablock(s) I. DOI: 10.1107/S160053681400347X/qm2104Isup2.hkl

Supporting information file. DOI: 10.1107/S160053681400347X/qm2104Isup3.cml

CCDC reference: 987315

Additional supporting information:  crystallographic information; 3D view; checkCIF report

## Figures and Tables

**Table 1:** Hydrogen-bond geometry (Å, °) *Cg*3 is the centroid of the C1–C6 benzene ring.

*D*—H⋯*A*	*D*—H	H⋯*A*	*D*⋯*A*	*D*—H⋯*A*
O1—H1*A*⋯Br2	0.85	2.49	3.332 (2)	170
O1—H1*B*⋯Br2^i^	0.85	2.61	3.271 (2)	135
N4—H4⋯O1	0.88	1.95	2.715 (3)	144
C15—H15⋯N2^ii^	0.95	2.62	3.571 (3)	177
C17—H17*B*⋯Br2^iii^	0.98	2.88	3.798 (3)	156
C20—H20⋯Br2^iv^	0.95	2.85	3.798 (3)	175
C21—H21⋯Br2^v^	0.95	2.92	3.579 (3)	127
C11—H11⋯*Cg*3^i^	0.95	2.93	3.789 (3)	152

Symmetry codes: (i) 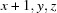; (ii) 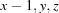; (iii) 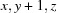; (iv) 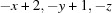; (v) 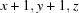.

**Fig. 1.**
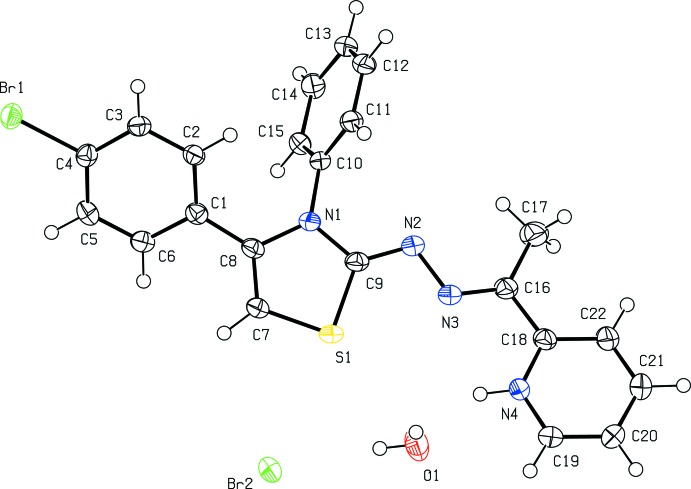
Perspective view of the asymmetric unit with 50% probability ellipsoids.

**Fig. 2.**
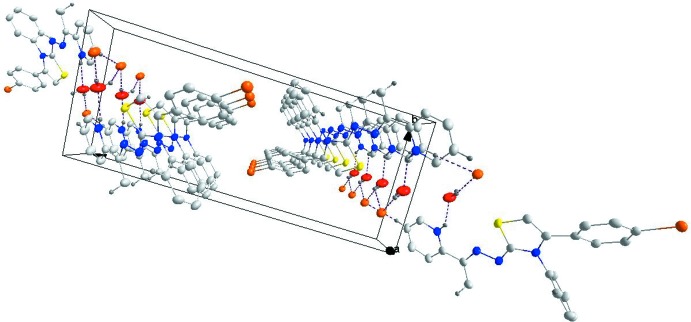
Packing viewed down the a axis showing the cation- anion-water chains with hydrogen bonds indicated by dotted lines.

**Fig. 3.**
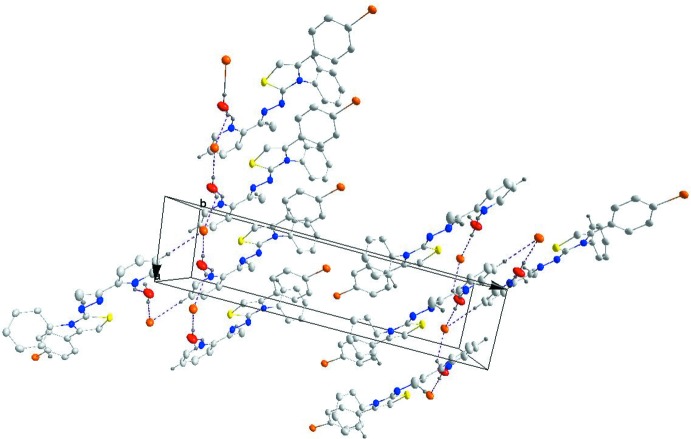
Packing viewed down the b axis giving a side view of the chains.

## supplementary crystallographic information

### 1. Comment

Thiazoles have shown a broad range of biological applications and activities (Siddiqui *et al.*, 2009; Quiroga *et al.*, 2002; Hutchinson, *et al*., 2002) including uses for the treatment of inflammation (Sharma *et al*., 2009), HIV infections (Bell *et al.*, 1995), hypertension (Patt *et al.*, 1992), schizophrenia (Jaen *et al.*, 1990), as hypnotics (Ergenc *et al.*, 1999), as fibrinogen receptor antagonists with antithrombotic activity (Badorc *et al.*, 1997) and as new inhibitors of bacterial DNA gyrase B (Rudolph *et al.*, 2001). In this context we report the synthesis and crystal structure of the title compound.

The N4/C18—C22, C1–C6 and C10–C15 aromatic rings make dihedral angles of 14.20 (12), 34.29 (10) and 68.75 (11)°, respectively, with the (S1/N1/C7–C9) thiazole ring (Fig. 1). The C9–N2–N3–C16, N2–N3–C16–C17, N2–N3–C16–C18 torsion angles are 174.0 (2), -4.0 (4) and 175.0 (5)°, respectively.

The three-dimensional structure of the title compound consists of chains running parallel to the *a* axis which are formed by association of the bromide ions and the lattice water molecules with the cations *via* O—H···N, O—H···Br, C—H···N and C—H···Br hydrogen bonding interactions (Table 1 and Figs. 2 & 3). In addition, a C—H···p interaction (Table 1) and a C4—Br1···*Cg*4 (-1 - *x*, 1 - *y*, 1 - *z*) interaction [Br1···*Cg*4 = 3.7426 (11) Å and C4—Br1···*Cg*4 = 161.73 (7)°] (Jasinski *et al.*, 2010; Zukerman-Schpector, *et al.*, 2011) also contribute to the stabilization of the molecular packing.

### 2. Experimental

A mixture of 270 mg (1 mmol) (2E)-*N*-phenyl-2-[1-(pyridin-2-yl)ethylidene]hydrazinecarbothioamide and 278 mg (1 mmol) 2-bromo-1-phenylethanone in absolute ethanol (30 ml) was refluxed for 8 h then cooled to room temperature. A yellow solid precipitated, it was filtered and washed with a small amount of cold ethanol and recrystallized from ethanol to afford good quality orange crystals (*M*.p. 521–523 K).

^1^H-NMR (CDCl_3_): δH= 1.63 (br, 1H, OH of H_2_O), 2.46 (s,3*H*, CH_3_), 6.41 (s, 1H, thiazole-CH), 6.97–7.02 (m, 2H, Ar—H), 7.21–7.23 (m, 2H, Ar—H), 7.54–7.61 (m, 1H, pyridine-CH), 7.72–7.79 (m, 1H, pyridine-CH), 8.22–8.25 (m, 2H, pyridine-CH), 9.10 (br, 1H, pyridinium-NH).

^13^C-NMR (CDCl_3_): δC= 14.02 (CH_3_), 104.27 (thiazole-CH), 127.89, 128.12, 128.36, 129.00, 129.92, 130.48, 131.77 (Ar—CH and pyridine-CH), 123.48, 131.98 (Ar—C), 142.04 (pridine-C), 153.27 (thiazole-C4), 156.38 (thiazole-C2).

### 3. Refinement

All H atoms were placed in geometrically idealized positions and constrained to ride on their parent atoms with O—H = 0.85 Å, N—H = 0.88 Å, C—H = 0.95 Å and 0.98 Å, with *U*_iso_(H) = 1.5 *U*_iso_(C) for methyl H atoms and *U*_iso_(H) = 1.2 *U*_iso_(C,*N*,*O*) for other H atoms.

### Crystal data

**Table:**

C_22_H_18_BrN_4_S^+^·Br^−^·H_2_O	*Z* = 2
*M**_r_* = 548.30	*F*(000) = 548
Triclinic, *P*1	*D*_x_ = 1.598 Mg m^−^^3^
Hall symbol: -P 1	Mo *K*α radiation, λ = 0.71073 Å
*a* = 5.5768 (6) Å	Cell parameters from 9919 reflections
*b* = 9.2288 (9) Å	θ = 2.4–29.1°
*c* = 22.574 (2) Å	µ = 3.69 mm^−^^1^
α = 85.974 (1)°	*T* = 150 K
β = 84.438 (1)°	Column, orange
γ = 79.000 (1)°	0.27 × 0.11 × 0.08 mm
*V* = 1133.51 (19) Å^3^	

### Data collection

**Table:**

Bruker SMART APEX CCD diffractometer	5870 independent reflections
Radiation source: fine-focus sealed tube	4807 reflections with *I* > 2σ(*I*)
Graphite monochromator	*R*_int_ = 0.038
φ and ω scans	θ_max_ = 29.3°, θ_min_ = 1.8°
Absorption correction: numerical (*SADABS*; Bruker, 2013)	*h* = −7→7
*T*_min_ = 0.390, *T*_max_ = 0.760	*k* = −12→12
20898 measured reflections	*l* = −30→30

### Refinement

**Table:**

Refinement on *F*^2^	Primary atom site location: structure-invariant direct methods
Least-squares matrix: full	Secondary atom site location: difference Fourier map
*R*[*F*^2^ > 2σ(*F*^2^)] = 0.035	H-atom parameters constrained
*wR*(*F*^2^) = 0.095	W = 1/[Σ^2^(*F*O^2^) + (0.0532*P*)^2^ + 0.0059*P*] WHERE *P* = (*F*O^2^ + 2*F*C^2^)/3
*S* = 1.09	(Δ/σ)_max_ < 0.001
5870 reflections	Δρ_max_ = 0.94 e Å^−^^3^
272 parameters	Δρ_min_ = −0.49 e Å^−^^3^
0 restraints	

### Special details

**Table:**

Experimental. The diffraction data were obtained from 3 sets of 400 frames, each of width 0.5° in ω, colllected at φ = 0.00, 90.00 and 180.00° and 2 sets of 800 frames, each of width 0.45° in φ, collected at ω = -30.00 and 210.00°. The scan time was 8 sec/frame.
Geometry. Bond distances, angles *etc*. have been calculated using the rounded fractional coordinates. All su's are estimated from the variances of the (full) variance-covariance matrix. The cell e.s.d.'s are taken into account in the estimation of distances, angles and torsion angles
Refinement. Refinement on *F*^2^ for ALL reflections except those flagged by the user for potential systematic errors. Weighted *R*-factors *wR* and all goodnesses of fit *S* are based on *F*^2^, conventional *R*-factors *R* are based on *F*, with *F* set to zero for negative *F*^2^. The observed criterion of *F*^2^ > σ(*F*^2^) is used only for calculating -*R*-factor-obs *etc*. and is not relevant to the choice of reflections for refinement. *R*-factors based on *F*^2^ are statistically about twice as large as those based on *F*, and *R*-factors based on ALL data will be even larger.

### Fractional atomic coordinates and isotropic or equivalent isotropic displacement parameters (Å^2^)

**Table:**

	*x*	*y*	*z*	*U*_iso_*/*U*_eq_	
Br1	−0.67572 (4)	0.25013 (3)	0.47615 (2)	0.0304 (1)	
S1	0.27834 (11)	0.54280 (7)	0.18810 (2)	0.0295 (2)	
N1	0.0981 (3)	0.6483 (2)	0.28912 (8)	0.0239 (5)	
N2	0.3783 (4)	0.7847 (2)	0.23756 (8)	0.0277 (6)	
N3	0.5417 (4)	0.7723 (2)	0.18707 (8)	0.0287 (6)	
N4	0.8686 (4)	0.7361 (2)	0.09224 (8)	0.0289 (6)	
C1	−0.1818 (4)	0.4718 (2)	0.33067 (10)	0.0244 (6)	
C2	−0.1555 (4)	0.4784 (2)	0.39151 (10)	0.0246 (6)	
C3	−0.3043 (4)	0.4146 (2)	0.43439 (10)	0.0254 (6)	
C4	−0.4804 (4)	0.3437 (2)	0.41671 (10)	0.0245 (6)	
C5	−0.5136 (4)	0.3367 (2)	0.35703 (10)	0.0276 (7)	
C6	−0.3638 (4)	0.4001 (2)	0.31457 (10)	0.0266 (6)	
C7	0.0640 (4)	0.4595 (3)	0.23186 (10)	0.0289 (7)	
C8	−0.0151 (4)	0.5258 (2)	0.28337 (10)	0.0247 (6)	
C9	0.2610 (4)	0.6731 (2)	0.24143 (9)	0.0253 (7)	
C10	0.0341 (4)	0.7538 (2)	0.33465 (9)	0.0223 (6)	
C11	0.2053 (4)	0.7667 (3)	0.37362 (10)	0.0265 (7)	
C12	0.1458 (4)	0.8746 (3)	0.41508 (10)	0.0303 (7)	
C13	−0.0796 (4)	0.9677 (3)	0.41774 (10)	0.0308 (7)	
C14	−0.2523 (4)	0.9507 (3)	0.37950 (11)	0.0302 (7)	
C15	−0.1951 (4)	0.8427 (2)	0.33767 (10)	0.0259 (6)	
C16	0.6559 (5)	0.8811 (3)	0.17393 (10)	0.0288 (7)	
C17	0.6171 (6)	1.0229 (3)	0.20592 (12)	0.0415 (9)	
C18	0.8431 (5)	0.8590 (3)	0.12317 (10)	0.0287 (7)	
C19	1.0414 (5)	0.7011 (3)	0.04748 (11)	0.0357 (8)	
C20	1.2093 (5)	0.7923 (3)	0.03072 (11)	0.0395 (8)	
C21	1.1876 (6)	0.9201 (3)	0.06046 (12)	0.0430 (9)	
C22	1.0058 (5)	0.9543 (3)	0.10605 (11)	0.0374 (8)	
Br2	0.27139 (5)	0.28139 (3)	0.09148 (2)	0.0383 (1)	
O1	0.7203 (4)	0.4762 (2)	0.08156 (10)	0.0527 (8)	
H2	−0.03430	0.52720	0.40350	0.0300*	
H3	−0.28530	0.41960	0.47550	0.0310*	
H4	0.77020	0.67170	0.09920	0.0350*	
H5	−0.63730	0.28920	0.34550	0.0330*	
H6	−0.38490	0.39500	0.27360	0.0320*	
H7	0.00780	0.37530	0.22060	0.0350*	
H11	0.36070	0.70280	0.37190	0.0320*	
H12	0.26180	0.88460	0.44200	0.0360*	
H13	−0.11680	1.04310	0.44560	0.0370*	
H14	−0.40940	1.01290	0.38190	0.0360*	
H15	−0.31250	0.83040	0.31150	0.0310*	
H17A	0.48760	1.02160	0.23850	0.0620*	
H17B	0.56860	1.10700	0.17790	0.0620*	
H17C	0.76970	1.03220	0.22220	0.0620*	
H19	1.04880	0.61340	0.02720	0.0430*	
H20	1.33540	0.76760	−0.00030	0.0470*	
H21	1.29900	0.98550	0.04950	0.0520*	
H22	0.99180	1.04350	0.12580	0.0450*	
H1A	0.59280	0.43750	0.08470	0.0630*	
H1B	0.81640	0.42050	0.10420	0.0630*	

### Atomic displacement parameters (Å^2^)

**Table:**

	*U*^11^	*U*^22^	*U*^33^	*U*^12^	*U*^13^	*U*^23^
Br1	0.0262 (1)	0.0324 (1)	0.0331 (1)	−0.0090 (1)	0.0018 (1)	−0.0010 (1)
S1	0.0374 (3)	0.0343 (3)	0.0186 (3)	−0.0109 (2)	0.0009 (2)	−0.0075 (2)
N1	0.0252 (10)	0.0272 (9)	0.0205 (9)	−0.0067 (8)	−0.0009 (7)	−0.0059 (7)
N2	0.0317 (11)	0.0306 (10)	0.0216 (9)	−0.0085 (8)	0.0009 (8)	−0.0043 (7)
N3	0.0340 (11)	0.0329 (10)	0.0211 (9)	−0.0106 (8)	−0.0008 (8)	−0.0049 (8)
N4	0.0347 (11)	0.0322 (10)	0.0237 (9)	−0.0165 (9)	−0.0011 (8)	−0.0027 (8)
C1	0.0233 (11)	0.0221 (10)	0.0274 (11)	−0.0021 (8)	−0.0021 (9)	−0.0042 (8)
C2	0.0233 (11)	0.0263 (11)	0.0252 (11)	−0.0055 (9)	−0.0013 (9)	−0.0069 (8)
C3	0.0253 (11)	0.0281 (11)	0.0225 (10)	−0.0032 (9)	−0.0011 (9)	−0.0048 (8)
C4	0.0207 (10)	0.0230 (10)	0.0283 (11)	−0.0018 (8)	0.0015 (9)	−0.0021 (8)
C5	0.0251 (11)	0.0251 (11)	0.0345 (12)	−0.0064 (9)	−0.0066 (9)	−0.0041 (9)
C6	0.0253 (11)	0.0288 (11)	0.0258 (11)	−0.0029 (9)	−0.0035 (9)	−0.0055 (9)
C7	0.0359 (13)	0.0313 (12)	0.0227 (11)	−0.0132 (10)	−0.0028 (9)	−0.0051 (9)
C8	0.0260 (11)	0.0271 (11)	0.0227 (10)	−0.0060 (9)	−0.0056 (8)	−0.0047 (8)
C9	0.0269 (12)	0.0301 (12)	0.0194 (10)	−0.0044 (9)	−0.0038 (8)	−0.0039 (8)
C10	0.0243 (11)	0.0236 (10)	0.0194 (10)	−0.0058 (8)	0.0012 (8)	−0.0043 (8)
C11	0.0208 (11)	0.0348 (12)	0.0247 (11)	−0.0053 (9)	−0.0023 (9)	−0.0054 (9)
C12	0.0295 (12)	0.0401 (13)	0.0243 (11)	−0.0118 (10)	−0.0020 (9)	−0.0087 (10)
C13	0.0337 (13)	0.0337 (12)	0.0275 (12)	−0.0120 (10)	0.0034 (10)	−0.0112 (9)
C14	0.0260 (12)	0.0258 (12)	0.0378 (13)	−0.0025 (9)	0.0009 (10)	−0.0059 (9)
C15	0.0253 (11)	0.0282 (11)	0.0260 (11)	−0.0075 (9)	−0.0045 (9)	−0.0029 (9)
C16	0.0384 (13)	0.0280 (11)	0.0214 (10)	−0.0098 (10)	−0.0034 (9)	0.0000 (9)
C17	0.0620 (19)	0.0294 (13)	0.0329 (13)	−0.0115 (12)	0.0041 (12)	−0.0037 (10)
C18	0.0389 (13)	0.0281 (11)	0.0216 (10)	−0.0115 (10)	−0.0057 (9)	−0.0004 (9)
C19	0.0446 (15)	0.0396 (14)	0.0264 (12)	−0.0169 (12)	0.0025 (11)	−0.0094 (10)
C20	0.0468 (16)	0.0459 (15)	0.0293 (13)	−0.0221 (13)	0.0086 (11)	−0.0063 (11)
C21	0.0525 (17)	0.0473 (16)	0.0358 (14)	−0.0298 (13)	0.0055 (12)	−0.0051 (12)
C22	0.0535 (17)	0.0340 (13)	0.0296 (13)	−0.0224 (12)	0.0020 (11)	−0.0033 (10)
Br2	0.0409 (2)	0.0393 (2)	0.0399 (2)	−0.0198 (1)	0.0017 (1)	−0.0117 (1)
O1	0.0430 (12)	0.0436 (12)	0.0761 (15)	−0.0216 (9)	0.0117 (10)	−0.0213 (10)

### Geometric parameters (Å, º)

**Table:**

Br1—C4	1.909 (2)	C13—C14	1.391 (3)
S1—C7	1.735 (2)	C14—C15	1.393 (3)
S1—C9	1.744 (2)	C16—C18	1.471 (4)
O1—H1A	0.8500	C16—C17	1.508 (4)
O1—H1B	0.8500	C18—C22	1.391 (4)
N1—C10	1.438 (3)	C19—C20	1.384 (4)
N1—C9	1.375 (3)	C20—C21	1.378 (4)
N1—C8	1.415 (3)	C21—C22	1.381 (4)
N2—C9	1.315 (3)	C2—H2	0.9500
N2—N3	1.386 (3)	C3—H3	0.9500
N3—C16	1.291 (3)	C5—H5	0.9500
N4—C19	1.339 (3)	C6—H6	0.9500
N4—C18	1.351 (3)	C7—H7	0.9500
N4—H4	0.8800	C11—H11	0.9500
C1—C2	1.402 (3)	C12—H12	0.9500
C1—C8	1.471 (3)	C13—H13	0.9500
C1—C6	1.401 (3)	C14—H14	0.9500
C2—C3	1.387 (3)	C15—H15	0.9500
C3—C4	1.382 (3)	C17—H17C	0.9800
C4—C5	1.385 (3)	C17—H17A	0.9800
C5—C6	1.383 (3)	C17—H17B	0.9800
C7—C8	1.349 (3)	C19—H19	0.9500
C10—C11	1.386 (3)	C20—H20	0.9500
C10—C15	1.379 (3)	C21—H21	0.9500
C11—C12	1.386 (4)	C22—H22	0.9500
C12—C13	1.379 (3)		
			
C7—S1—C9	90.19 (11)	N4—C18—C22	116.9 (2)
H1A—O1—H1B	104.00	N4—C19—C20	120.2 (2)
C8—N1—C10	126.06 (18)	C19—C20—C21	117.8 (3)
C9—N1—C10	119.83 (17)	C20—C21—C22	120.7 (3)
C8—N1—C9	113.57 (17)	C18—C22—C21	120.4 (2)
N3—N2—C9	108.59 (17)	C3—C2—H2	120.00
N2—N3—C16	116.19 (19)	C1—C2—H2	120.00
C18—N4—C19	123.9 (2)	C2—C3—H3	120.00
C19—N4—H4	113.00	C4—C3—H3	120.00
C18—N4—H4	123.00	C6—C5—H5	121.00
C2—C1—C6	118.1 (2)	C4—C5—H5	121.00
C6—C1—C8	118.7 (2)	C5—C6—H6	119.00
C2—C1—C8	123.0 (2)	C1—C6—H6	119.00
C1—C2—C3	120.8 (2)	S1—C7—H7	123.00
C2—C3—C4	119.4 (2)	C8—C7—H7	123.00
C3—C4—C5	121.4 (2)	C10—C11—H11	121.00
Br1—C4—C5	119.76 (16)	C12—C11—H11	121.00
Br1—C4—C3	118.87 (17)	C13—C12—H12	120.00
C4—C5—C6	118.9 (2)	C11—C12—H12	120.00
C1—C6—C5	121.5 (2)	C12—C13—H13	120.00
S1—C7—C8	113.51 (19)	C14—C13—H13	120.00
N1—C8—C7	111.7 (2)	C15—C14—H14	120.00
N1—C8—C1	123.17 (18)	C13—C14—H14	120.00
C1—C8—C7	124.91 (19)	C10—C15—H15	120.00
N1—C9—N2	122.65 (18)	C14—C15—H15	120.00
S1—C9—N2	126.31 (16)	C16—C17—H17B	110.00
S1—C9—N1	111.01 (15)	C16—C17—H17C	109.00
C11—C10—C15	121.3 (2)	H17A—C17—H17B	109.00
N1—C10—C11	119.52 (19)	H17A—C17—H17C	109.00
N1—C10—C15	119.14 (19)	H17B—C17—H17C	109.00
C10—C11—C12	118.8 (2)	C16—C17—H17A	109.00
C11—C12—C13	120.8 (2)	N4—C19—H19	120.00
C12—C13—C14	119.7 (2)	C20—C19—H19	120.00
C13—C14—C15	120.1 (2)	C21—C20—H20	121.00
C10—C15—C14	119.2 (2)	C19—C20—H20	121.00
C17—C16—C18	118.8 (2)	C20—C21—H21	120.00
N3—C16—C18	114.8 (2)	C22—C21—H21	120.00
N3—C16—C17	126.3 (2)	C18—C22—H22	120.00
N4—C18—C16	119.0 (2)	C21—C22—H22	120.00
C16—C18—C22	124.0 (2)		
			
C9—S1—C7—C8	−0.05 (19)	C2—C1—C8—C7	141.2 (2)
C7—S1—C9—N1	0.22 (17)	C6—C1—C8—N1	151.9 (2)
C7—S1—C9—N2	−177.7 (2)	C6—C1—C8—C7	−33.7 (3)
C9—N1—C8—C1	175.39 (19)	C1—C2—C3—C4	0.0 (3)
C9—N1—C8—C7	0.3 (3)	C2—C3—C4—Br1	177.72 (15)
C10—N1—C8—C1	−13.1 (3)	C2—C3—C4—C5	−0.9 (3)
C10—N1—C8—C7	171.8 (2)	Br1—C4—C5—C6	−177.46 (15)
C8—N1—C9—S1	−0.3 (2)	C3—C4—C5—C6	1.1 (3)
C8—N1—C9—N2	177.7 (2)	C4—C5—C6—C1	−0.5 (3)
C10—N1—C9—S1	−172.41 (15)	S1—C7—C8—N1	−0.1 (2)
C10—N1—C9—N2	5.6 (3)	S1—C7—C8—C1	−175.12 (18)
C8—N1—C10—C11	117.6 (2)	N1—C10—C11—C12	176.4 (2)
C8—N1—C10—C15	−63.9 (3)	C15—C10—C11—C12	−2.0 (3)
C9—N1—C10—C11	−71.4 (3)	N1—C10—C15—C14	−176.3 (2)
C9—N1—C10—C15	107.1 (2)	C11—C10—C15—C14	2.1 (3)
C9—N2—N3—C16	174.0 (2)	C10—C11—C12—C13	0.1 (4)
N3—N2—C9—S1	−7.0 (3)	C11—C12—C13—C14	1.8 (4)
N3—N2—C9—N1	175.30 (19)	C12—C13—C14—C15	−1.7 (4)
N2—N3—C16—C17	−4.0 (4)	C13—C14—C15—C10	−0.2 (3)
N2—N3—C16—C18	175.0 (2)	N3—C16—C18—N4	4.8 (4)
C19—N4—C18—C16	−176.2 (2)	N3—C16—C18—C22	−172.3 (2)
C19—N4—C18—C22	1.1 (4)	C17—C16—C18—N4	−176.2 (2)
C18—N4—C19—C20	0.6 (4)	C17—C16—C18—C22	6.8 (4)
C6—C1—C2—C3	0.5 (3)	N4—C18—C22—C21	−1.7 (4)
C8—C1—C2—C3	−174.40 (18)	C16—C18—C22—C21	175.4 (3)
C2—C1—C6—C5	−0.3 (3)	N4—C19—C20—C21	−1.6 (4)
C8—C1—C6—C5	174.87 (18)	C19—C20—C21—C22	0.9 (4)
C2—C1—C8—N1	−33.2 (3)	C20—C21—C22—C18	0.8 (4)

### Hydrogen-bond geometry (Å, º)

Cg3 is the centroid of the C1–C6 benzene ring.

**Table:**

*D*—H···*A*	*D*—H	H···*A*	*D*···*A*	*D*—H···*A*
O1—H1*A*···Br2	0.85	2.49	3.332 (2)	170
O1—H1*B*···Br2^i^	0.85	2.61	3.271 (2)	135
N4—H4···O1	0.88	1.95	2.715 (3)	144
C15—H15···N2^ii^	0.95	2.62	3.571 (3)	177
C17—H17*A*···N2	0.98	2.38	2.798 (4)	105
C17—H17*B*···Br2^iii^	0.98	2.88	3.798 (3)	156
C19—H19···O1	0.95	2.59	3.006 (3)	107
C20—H20···Br2^iv^	0.95	2.85	3.798 (3)	175
C21—H21···Br2^v^	0.95	2.92	3.579 (3)	127
C11—H11···*Cg*3^i^	0.95	2.93	3.789 (3)	152

Symmetry codes: (i) *x*+1, *y*, *z*; (ii) *x*−1, *y*, *z*; (iii) *x*, *y*+1, *z*; (iv) −*x*+2, −*y*+1, −*z*; (v) *x*+1, *y*+1, *z*.

## References

[bb1] Badorc, A., Bordes, M. F., De Cointet, P., Savi, P., Bernat, A., Lale, A., Petitou, M., Maffrand, J. P. & Herbert, J. M. (1997). *J. Med. Chem.* **40**, 3393–3401. 10.1021/jm970240y 9341914

[bb2] Bell, F. W., Cantrell, A. S., Hogberg, M., Jaskunas, S. R., Johansson, N. G., Jordon, C. L., Kinnick, M. D., Lind, P., Morin, J. M., Noreen, R., Oberg, B., Palkowitz, J. A., Parrish, C. A., Pranc, P., Sahlberg, C., Ternansky, R. J., Vasileff, R. T., Vrang, L., West, S. J., Zhang, H. & Zhou, X. X. (1995). *J. Med. Chem.* **38**, 4929–4936. 10.1021/jm00025a010 8523406

[bb3] Brandenburg, K. & Putz, H. (2012). *DIAMOND* Crystal Impact GbR, Bonn, Germany.

[bb4] Bruker (2013). *APEX2*, *SAINT* and *SADABS* Bruker AXS Inc., Madison, Wisconsin, USA.

[bb5] Ergenc, N., Capan, G., Gunay, N. S., Ozkirimli, S., Gungor, M., Ozbey, S. & Kendi, E. (1999). *Arch. Pharm. Pharm. Med. Chem.* **332**, 343–347. 10.1002/(sici)1521-4184(199910)332:10<343::aid-ardp343>3.0.co;2-0 10575366

[bb6] Farrugia, L. J. (2012). *J. Appl. Cryst.* **45**, 849–854.

[bb7] Hutchinson, I., Jennings, S. A., Vishnuvajjala, B. R., Westwell, A. D. & Stevens, M. F. G. (2002). *J. Med. Chem.* **45**, 744–747. 10.1021/jm011025r 11806726

[bb8] Jaen, J. C., Wise, L. D., Caprathe, B. W., Tecle, H., Bergmeier, S., Humblet, C. C., Heffner, T. G., Meltzner, L. T. & Pugsley, T. A. (1990). *J. Med. Chem.* **33**, 311–317. 10.1021/jm00163a051 1967314

[bb9] Jasinski, J. P., Guild, C. J., Samshuddin, S., Narayana, B. & Yathirajan, H. S. (2010). *Acta Cryst.* E**66**, o2018. 10.1107/S1600536810026905 PMC3007578 21588329

[bb10] Patt, W. C., Hamilton, H. W., Taylor, M. D., Ryan, M. J., Taylor, D. J., Connolly, C. J. C., Doherty, A. M., Klutchko, S. R., Sircar, I., Steinbaugh, B. A., Batley, B. L., Painchaud, C. A., Rapundalo, S. T., Michniewicz, B. M. & Olson, S. C. J. (1992). *J. Med. Chem.* **35**, 2562–2572. 10.1021/jm00092a006 1635057

[bb11] Quiroga, J., Hernandez, P., Insuasty, B., Abonia, R., Cobo, J., Sanchez, A., Nogueras, M. & Low, J. N. (2002). *J. Chem. Soc. Perkin Trans. 1*, **4**, 555–559.

[bb12] Rudolph, J., Theis, H., Hanke, R., Endermann, R., Johannsen, L. & Geschke, F. U. (2001). *J. Med. Chem.* **44**, 619–626. 10.1021/jm0010623 11170652

[bb13] Sharma, R. N., Xavier, F. P., Vasu, K. K., Chaturvedi, S. C. & Pancholi, S. S. (2009). *J. Enzyme Inhib. Med. Chem.* **24**, 890–897. 10.1080/14756360802519558 19469712

[bb14] Sheldrick, G. M. (2008). *Acta Cryst.* A**64**, 112–122. 10.1107/S0108767307043930 18156677

[bb15] Siddiqui, N., Arshad, M. F., Ahsan, W. & Alam, M. S. (2009). *IJPSDR*, **1**, 136–143.

[bb16] Spek, A. L. (2009). *Acta Cryst.* D**65**, 148–155. 10.1107/S090744490804362X PMC2631630 19171970

[bb17] Zukerman-Schpector, J., De Simone, C. A., Olivato, P. R., Cerqueira, C. R. & Tiekink, E. R. T. (2011). *Acta Cryst.* E**67**, o1099–o1100. 10.1107/S1600536811012712 PMC3089188 21754419

